# Assessing the MODIS Crop Detection Algorithm for Soybean Crop Area Mapping and Expansion in the Mato Grosso State, Brazil

**DOI:** 10.1155/2014/863141

**Published:** 2014-04-10

**Authors:** Anibal Gusso, Damien Arvor, Jorge Ricardo Ducati, Mauricio Roberto Veronez, Luiz Gonzaga da Silveira

**Affiliations:** ^1^Environmental Engineering, Vale do Rio dos Sinos University (UNISINOS), CP 275, São Leopoldo, RS, Brazil; ^2^Center for Remote Sensing and Meteorological Research, Federal University of Rio Grande do Sul (UFRGS), 15044 Porto Alegre, RS, Brazil; ^3^VizLab—Advanced Visualization Laboratory, Vale do Rio dos Sinos University (UNISINOS), São Leopoldo, Brazil; ^4^IRD-UMR 228 ESPACE-DEV (IRD, UM2, UAG, UR), MTD-Montpellier, 500 rue Jean-François Breton, 34093 Montpellier Cedex, France; ^5^Astronomy Department, Federal University of Rio Grande do Sul (UFRGS), 15051 Porto Alegre, RS, Brazil; ^6^Graduate Program in Geology, Vale do Rio dos Sinos University (UNISINOS), CP 275, São Leopoldo, RS, Brazil; ^7^Graduate Program in Applied Computing, Vale do Rio dos Sinos University (UNISINOS), CP 275, São Leopoldo, RS, Brazil

## Abstract

Estimations of crop area were made based on the temporal profiles of the Enhanced Vegetation Index (EVI) obtained from moderate resolution imaging spectroradiometer (MODIS) images. Evaluation of the ability of the MODIS crop detection algorithm (MCDA) to estimate soybean crop areas was performed for fields in the Mato Grosso state, Brazil. Using the MCDA approach, soybean crop area estimations can be provided for December (first forecast) using images from the sowing period and for February (second forecast) using images from the sowing period and the maximum crop development period. The area estimates were compared to official agricultural statistics from the Brazilian Institute of Geography and Statistics (IBGE) and from the National Company of Food Supply (CONAB) at different crop levels from 2000/2001 to 2010/2011. At the municipality level, the estimates were highly correlated, with *R* ^2^ = 0.97 and RMSD = 13,142 ha. The MCDA was validated using field campaign data from the 2006/2007 crop year. The overall map accuracy was 88.25%, and the Kappa Index of Agreement was 0.765. By using pre-defined parameters, MCDA is able to provide the evolution of annual soybean maps, forecast of soybean cropping areas, and the crop area expansion in the Mato Grosso state.

## 1. Introduction

Crop monitoring is a major concern for food safety and the regulation of the agricultural market. Many programs have been established by agricultural agencies to regularly provide agricultural statistics at different spatial and temporal scales (e.g., the MARS project in Europe or GeoSafras in Brazil). The GEO-GLAM (global agriculture monitoring) project is working to harmonize remote sensing-based crop monitoring systems. In that context, the case of Brazil remains atypical. Brazil is currently considered to be one of the world's granaries and plays an important role in global markets as a main producer of agricultural commodities. However, official agricultural statistics released by two Brazilian agencies, namely, Companhia Nacional de Abastecimento—National Company of Food Supply (CONAB) and Instituto Brasileiro de Geografia e Estatística—Brazilian Institute of Geography and Statistics (IBGE), suffer from two main issues. (1) Municipality statistics are not released shortly after harvest, but rather they are released nearly 18 months after the end of the soybean season; and (2) there is a lack of confidence in the production estimates because they are based on subjective methods and are associated with error measurements [1–3].

Remote sensing data has the potential to address these issues because enhanced temporal resolution allows producing near-real-time estimates of agricultural statistics. In Brazil, several studies led by governmental and nongovernmental organizations have focused on crop mapping and forecasting [3–5]. However, most of these studies were designed for a few cropping years and/or for a limited region. For example, remote sensing images (such as Landsat TM or CBERS) have been used for mapping sugarcane in the CANASAT project by INPE (Instituto Nacional de Pesquisas Espaciais—Brazilian Space Agency) and for mapping soybean in the GeoSafras Project by CONAB. Although these projects confirmed the efficiency of satellite images for mapping perennial and semiperennial crops, the monitoring of annual crops, such as soybean, corn, or cotton, remains an issue. The high incidence of cloud cover during key identification periods of annual crops and the 16-day temporal resolution hindered the operational implementation of Landsat- or CBERS-based methodologies for calculating agricultural statistics [6, 7].

Overcoming the cloud cover challenge requires an increased temporal resolution of the orbital sensors, often at the expense of the spatial resolution. The moderate resolution imaging spectroradiometer (MODIS) sensor on board of the Terra satellite provides an adequate imaging configuration for crop monitoring based on (1) an almost-daily revisit time; (2) a moderate spatial resolution of 250 m, considered adequate for mapping large-scale agricultural fields [8]; and (3) geometric quality that is high enough for image time series analysis [9].

In the USA, the quality of MODIS data was evaluated for its potential to provide information on both crop yield and crop area [10]. In another study [11], the applicability of MODIS/EVI time series data for mapping agricultural lands was investigated; the study concluded that 16-day composites of MODIS images gave sufficient spatial, spectral, and temporal information to perform the following: (1) adequately separate crop fields from other land uses and (2) express the phenology and climate characteristics of the region. In Brazil, many works have highlighted the efficiency of MODIS time series of vegetation indices for mapping croplands, crop expansion [12, 13], and cropping systems [14–16].

Although all studies have confirmed the potential of MODIS sensors for crop mapping, a few challenges remain to be solved to confirm its role as an alternative to traditional official agricultural estimate methods. First, most MODIS-based analyses were tested and validated at a local or state scale, and their validity for mapping crops in other agricultural areas remains uncertain. For example, many classification methods consist of supervised approaches based on training samples, which implies the following: (1) it is laborious and costly to get training samples for large-scale areas and (2) agricultural calendars vary drastically between different agricultural areas and over time, especially in frontier areas such as the Amazon, where agricultural practices are evolving rapidly [15, 17–19]. These challenges have hindered the use of vegetation index time series for large-scale crop mapping. Consequently, implementing an operational system at a nation-wide scale represents a huge challenge; it requires the development of a robust method that accounts for the spatial variability of environmental conditions and agricultural practices across Brazil. Second, most classification systems are based on a complete vegetation index time series, a process that makes the production of near-real-time or forecast estimates slow or difficult, which reduces the benefits of using remote sensing data compared with traditional agricultural statistics.

In the present paper, we argue that an operational crop monitoring model should be (1) adapted to specific regional agricultural calendars and (2) based on subsets of vegetation time series to allow an early release of agricultural statistics. To evaluate this hypothesis, we assessed the efficiency of the MODIS crop detection algorithm (MCDA) as proposed by Gusso et al. (2012) [1], which is an example of an operational crop monitoring model. This method has been initially validated for mapping soybean crops in southern Brazil (state of Rio Grande do Sul), and we now demonstrate its efficiency for crop mapping in the Amazonian state of Mato Grosso, which is characterized by different environmental conditions and agricultural practices. MCDA was validated here in a completely different region (southern Amazon) with different crop calendars, double cropping systems that might affect the accuracy of the model for detecting soybean crops and intense spatial dynamics because soybean cropping has expanded significantly in the last decade.

## 2. Materials and Methods

### 2.1. Study Area

**Figure 1:**
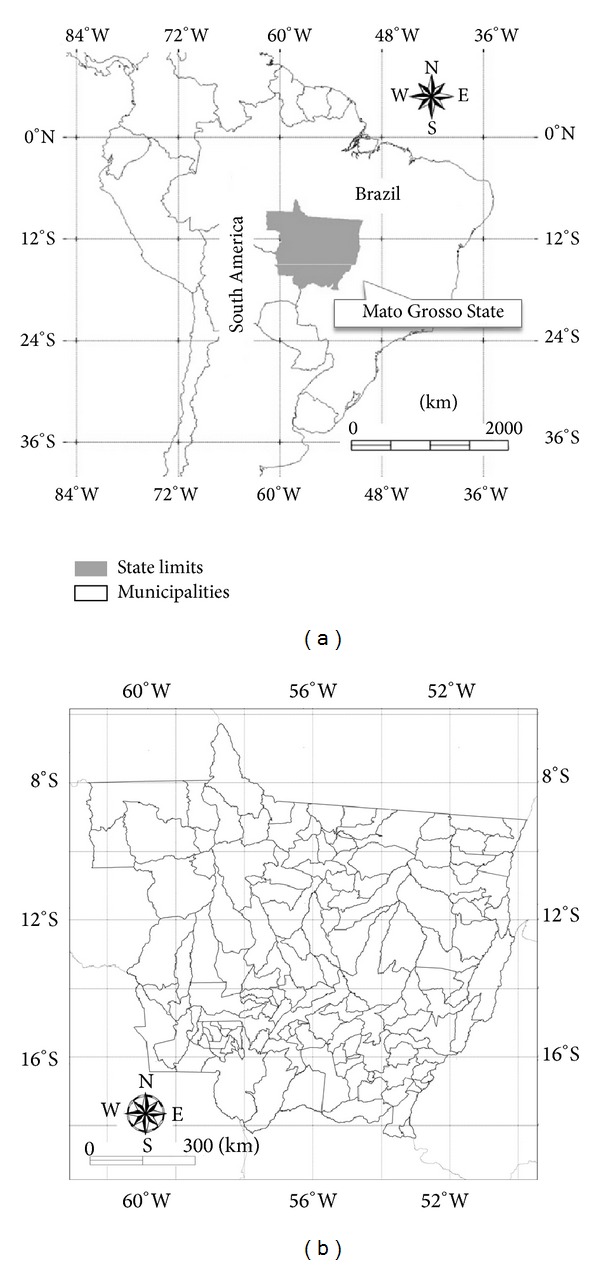
Mato Grosso State in Brazil and its 146 municipalities.

The state of Mato Grosso (906,000 km², 146 municipalities) is located in the southern portion of the Amazon basin and is characterized by three main biomes: the Brazilian Cerrado, the Amazon rainforest, and the Pantanal. Since the 1970s, a crop expansion process led Mato Grosso to be the largest soybean producer in Brazil (approximately 30% of the national soybean production occurs in Mato Grosso) [20]. In the last decade, intensive practices, such as double cropping, have been widely adopted in Mato Grosso, which is an additional challenge for accurate crop area mapping [18]. Usually, soybean remains the main crop, while maize or cotton is planted after the soybean harvest [15]. Typically, the sowing period for soybean lasts from mid-September to late October and depends mainly on the sowing dates, which are determined according to the onset of the rainy season, which lasts from October to May; the area of study is presented in Figure 1.

### 2.2. Materials

#### 2.2.1. Input Data for Applying the MCDA Procedure

We used several datasets to represent the main physical conditions and management practices found in Mato Grosso. First, we acquired MODIS EVI data (MOD13Q1 product, collections 5 and 6) covering all of the Mato Grosso state (image tiles: H11V09, H11V10, H12V09, H12V10, H13V09, and H13V10) for the 2000–2011 study period. The EVI data were chosen for their potential to mitigate cloud cover effects and atmospheric and soil background effects [9, 21]. The EVI data are a 16-day composite with high radiometric and geometric corrections. The MODIS images and products were preprocessed by the National Aeronautics and Space Administration (NASA) and are available at no charge at https://lpdaac.usgs.gov/data_access/data_pool.

Second, Shuttle Radar Topography Mission (SRTM) data [22] were used to generate a slope map with a 90-meter spatial resolution according to the method described by Gusso et al. [1]. This map was used to exclude areas inappropriate for mechanization (slope > 12%); soybean in Mato Grosso is a highly mechanized crop and requires relatively smooth land to allow the use of farm machinery [23].

Third, we acquired 10-day accumulated precipitation data from September to October at 11 meteorological stations for each year of the study period (2000–2011) to determine the initial sowing period. These data were acquired from Instituto Nacional de Meteorologia—Brazilian Institute of Meteorology (INMET).

#### 2.2.2. Validation Data

Two types of datasets were acquired to validate our approach at different scales, that is, state- and municipality-level datasets and pixel-level datasets. First, we used annual soybean agricultural statistics at the state and municipality levels from [20, 24] for the entire study area. Second, a field campaign was carried out in 2006 and 2007 to collect validation data at the crop field scale. A total of 76 farms were visited and mapped in 13 municipalities representing the two main agricultural regions (in central and western Mato Grosso, along the BR163 road and on the Chapada dos Parecis, resp.). A complete map of the visited municipalities is introduced in [15]. For each farm, information about crop types, yields, sowing, and harvesting dates were collected. In this study, we only considered the crop type information for the 2006/2007 season's harvest. For that specific cropping season, only 38 farms were considered (information on other farms were not available for that season), representing 1,078 fields for a total area of 196,929 ha (i.e., a total of 31,508 MODIS pixels and a mean field area of 182.7 ha).

### 2.3. MCDA Calibration for Mato Grosso

**Figure 2:**
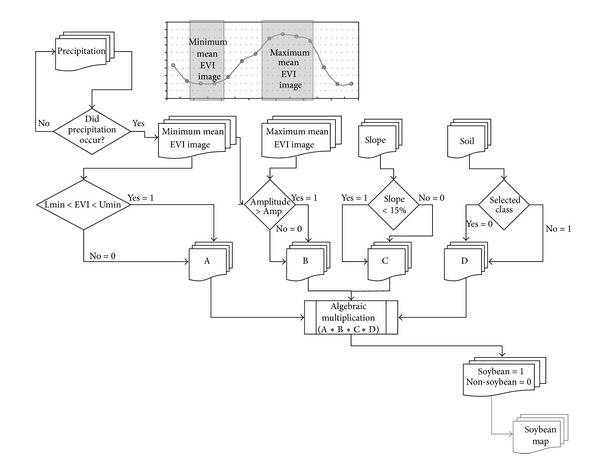
Flowchart of the MCDA classification [1] based on MODIS/EVI images.

The MODIS crop detection algorithm (MCDA) was used to classify soybean crops in this study; a diagrammatic flowchart is presented in Figure 2. This procedure classifies a pixel as soybean if it adheres to conditions A and B in Figure 2. Conditions A and B are related to the regional soybean calendar and vegetation development characteristics starting from the sowing period. Conditions C and D are related to terrain characteristics and management, which are not expected to vary from one cropping year to another. The present work aimed to adapt and to test the MCDA; specifically, we evaluated how MCDA adheres to conditions A and B described in [1]. To establish these two conditions, three parameters need to be defined: Amp (amplitude, which is the difference between maximum and minimum EVI values); Lmin (the lower minimum EVI value in a minimum image), and Umin (the maximum EVI value in a minimum image). These parameters are defined based on the analysis of the observed EVI time series for cropping areas. The time series are characterized by low EVI values during the presowing period (September-October) and high EVI values during the maximum crop development period (January-February) [15]. Adjustments of MCDA parameters will be referred to as the MCDA calibration.

The set of initial parameters of three test sites of 100 × 100 pixels in Mato Grosso were obtained from MODIS/EVI images; the parameters are from two specific periods for each crop year according to the methodology developed in [1]. The periods are as follows: sowing (day of year (DOY) 225 to 337) and maximum crop development (DOY 353 to 033). The sowing period often starts in September, but the beginning of the sowing period is determined by the rainfall in each crop year, in agreement with the soybean zoning provided by [25].

**Figure 3:**
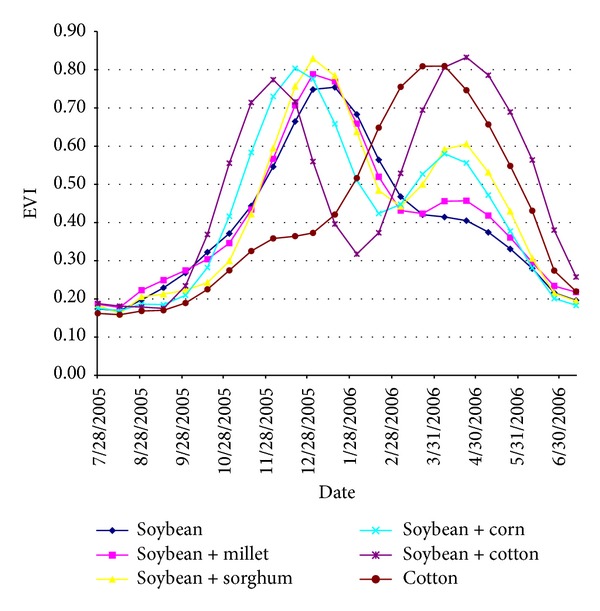
Mean MODIS/EVI time series for selected agricultural plots [15].

Figure 3 presents the mean EVI time series acquired over the crop fields that coexist. The natural vegetation cover in the region is typical of the Cerrado biome; this vegetation cover causes some confusion with detecting soybean development during the rainy season [3, 15]. For the EVI time series, two major classes can be identified: single and double cropping systems. Single cropping systems refer to soybean or cotton that is sown without any other crop being planted before or after the main crop. Soybean and cotton can thus be differentiated based on the agricultural calendar because cotton is sown in December and harvested in June. For double cropping systems, another crop (usually cotton, maize, millet, or sorghum) is sown after the soybean harvest.

Two MODIS/EVI images from the sowing period are first averaged two by two to obtain the minimum mean EVI image (MinMeanEVI), which smoothens the EVI profile for a relatively short time window. This MinMeanEVI image then defines the Lmin and Umin parameters, that is, the lower and upper EVI values for each cropping year, respectively. Pixels below Lmin are typically associated with cloud shadows or water bodies. According to the methodology developed in [1], Umin is set as the convergence between the minimum and maximum mean EVI images, and pixels above Umin are not from annual crops. Pixels with EVI values between Lmin and Umin are designated as soybean crop pixels in accordance with condition A in Figure 2.

During the sowing period, increasing MODIS/EVI values are observed because of rapid and intense plant growing; maximum values are reached after a relatively short period [11]. To characterize the maximum mean EVI image (MaxMeanEVI), four consecutive EVI images from this period (DOY 353 to 033; Figure 2) are averaged. The difference between MaxMeanEVI and MinMeanEVI is computed to produce the EVI amplitude image (AmpEVI) for each cropping year, according to the procedure outlined in [1]. However, the remaining challenge is to obtain the best Amp value that includes not only pure soybean pixels (with high values in the maximum EVI image and low values in the minimum EVI image) but also mixed pixels located at the border of the soybean fields. The optimal Amp value for each cropping year can be obtained from the convergence region between the minimum and maximum EVI values in the scatterplots, as described in [1]. The Amp value is the minimum difference between the maximum and the minimum mean EVI values to which a mixed soybean pixel can be designated as a soybean pixel. Pixels with amplitude values greater than Amp are tagged as soybean according to condition B of the MCDA procedure (Figure 2).

The soybean area can be estimated after the maximum mean EVI image (MaxMeanEVI) is available, which normally occurs in January, because the MCDA approach uses the half-phase of the crop development. The MOD13Q1 product is often available after a delay of approximately 20 days. Therefore, the soybean estimation should be released no later than early February (from now on referred as the second forecast of the MCDA). However, as an alternative to forecasting the soybean area, a first estimate can be provided in early December of each crop year based on the MinMeanEVI image. This image is strongly related to the sowing period of the current crop year, and the MaxMeanEVI image of a previous crop year (referred to as the first forecast of the MCDA). The same procedure as the first forecast can be performed if no usable images were found or if a water deficit is empirically observed (30 days without a rainfall event over 10 mm); in that case, images from previous normal crop years are used to generate the maximum mean EVI image. However, for crop year 2000/2001, no first forecast of MCDA was available because there were no MODIS data before 2000.

### 2.4. Adjustment of Management Practices

MCDA was developed to provide an objective and automated tool for soybean classification. The MCDA accuracy was validated in one step. However, the MCDA calibration procedure is not complete until the same Mato Grosso input parameters, which were chosen to represent the physically driven components defined in the MCDA, can be used for all of the evaluated crop years. Therefore, once identified, the parameters from the physically driven components cannot be adjusted after the fact; this constrains the dynamical adjustment process of the algorithm [1]. If some further adjustment is needed to improve the fit of the crop areas with statistics from IBGE for one or more crop years, then this new parameter value must be used for all of the tested crops. After several iterations for all crop years, the adjusted combination with the best performance to define the final values of MCDA was found to be 0.05, 0.39, and 0.36 for Umin, Lmin, and Amp, respectively.

## 3. Results

**Figure 4:**
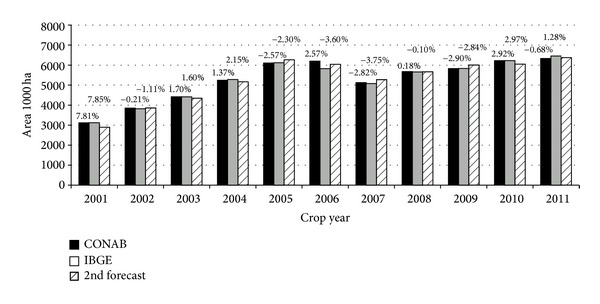
Comparison of the MCDA (second forecast), IBGE, and CONAB soybean area estimates for the Mato Grosso State and percent difference from MCDA.

Based on the previously described analysis, the soybean area was estimated at the municipality level for 141 municipalities in Mato Grosso state from 2000/2001 to 2010/2011 and was compared to the official estimates provided at http://www.sidra.ibge.gov.br/ [20] using a regression analysis. The MCDA soybean area estimates are provided at state and municipal levels due to the spatial distribution of the classified soybean area. Therefore, these estimates can be compared to the IBGE and CONAB municipal statistics; comparison of the MCDA, IBGE, and CONAB area estimates are presented in Figure 4. No prior year estimate could be provided for the 2000/2001 because this was the first crop year in which MODIS data became available. No further MCDA maps were generated after 2010/2011 because the data from crop years 2011/2012 and 2012/2013 had not yet been released by IBGE at the municipal level.

### 3.1. State Area Estimates

The MCDA second forecast estimates for Mato Grosso were compared to IBGE and CONAB statistics. The maximum difference observed across the study period was 7.85% above MCDA for the 2000/2001 crop season.

### 3.2. Municipality Area Estimates

**Figure 5:**
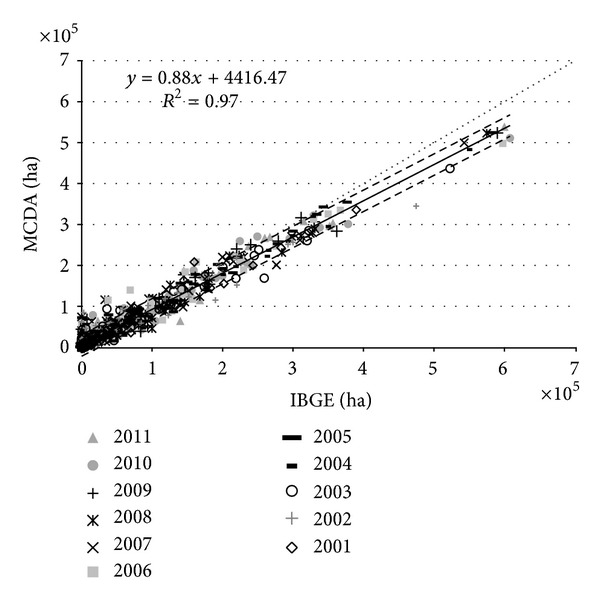
Regression analysis between soybean area estimates by MCDA (second forecast) and IBGE for the Mato Grosso State for all crop years (2000/2001 to 2010/2011).

Soybean area was estimated at the municipality level from the crop season 2000/2001 to 2010/2011 and was compared to official estimates provided by IBGE http://www.sidra.ibge.gov.br/ using a regression analysis. Quickly updating the current crop year is challenging because the IBGE municipality data are published approximately one year after the end of the soybean season.  Figure 5 presents the linear least squares regression analysis for the municipal soybean estimates from the MCDA procedure and from IBGE for crop years 2000/2001 to 2010/2011 with *R* ^2^ = 0.97. Therefore, for the average of the eleven crop years studied, the MCDA explains 97% of the variation of the data estimated by IBGE, which indicates a good agreement between the estimates. The group of points above 500,000 ha represents the crop area of Sorriso municipality, which had 483,000 ha of soybean crop area in 2004 [20]. Overall, the MCDA results slightly underestimated the soybean crop area in comparison to municipal data from IBGE. The intercept value in the overall linear regression was 4416 ha, which indicates that municipalities with small soybean areas are slightly overestimated by the MCDA in comparison to the IBGE estimates. The positive intercept value indicates that, in general, the overestimated municipalities are typically <50,000 ha. The slope value in the overall linear regression was 0.88. The root mean square deviation (RMSD) for the second forecast of the MCDA was approximately 13,142 ha for all crop years; the thin dashed lines are the double RMSD (above and below the regression line) and contain 95% of the data points. USA soybean area estimates from [26] were generated by MODIS and by the USDA/NASS, and for these estimates, the *R* ^2^ values ranged from 0.44 to 0.94 and the RMSD varied from 41,465 to 120,955 ha for the entire USA.

### 3.3. Crop Level Estimates

The 2006-2007 MCDA crop map was compared to the field data collected during that specific harvest to validate the classification accuracy. Based on the field data introduced in Section 2.2.2, we randomly selected 400 MODIS pixels (200 soybean pixels and 200 non-soybean pixels).  Table 1 shows the confusion matrix resulting from the comparison between these randomly selected pixels and the crop map produced by MCDA for the cropping year 2006/2007. The overall map accuracy was approximately 88.25%, and the Kappa Index of Agreement was 0.765, a satisfactory value, because for the assessment of classification maps, Kappa values greater than 0.5 are considered satisfactory [27]. Soybean area estimates from [28], generated by MODIS and by the USDA/NASS for different ecoregions in the Great Lakes—USA, obtained overall accuracy of 82%.

## 4. Discussion

The MCDA model was developed with a focus on soybean crop area identification. However, the validation of large area mapping still presents a difficult challenge [28]. The MCDA approach establishes the input parameters as fixed criteria; therefore, the same input parameters are used independent of crop year dynamics during the evaluated period, from 2000/2001 to 2010/2011. This is an important consideration for the analysis of the 80.97% producer's accuracy result.

According to field campaign data used for the crop level evaluation of MCDA, the resulting user's accuracy, almost 100%, strongly indicates that the MCDA detection of soybean crop area is reliable. However, the 80.97% obtained from producers' accuracy analysis is related to 47 misidentifications highlighted in Section 3.3, and this result deserves further analysis.

Direct visual inspection of the data indicates that the pixels misidentifications presented in Table 1 can be explained as follows: the majority of the cases in the 2006/2007 crop (31 over 47 pixels) came from double crop system practices in which cotton came after soybean. As cotton is a valuable commodity and tends to have a longer cycle, it is worthwhile for the producer to plan the soybean cycle to provide sufficient time for the cotton crop (as seen in Figure 3). According to the summer crop calendar, this schedule shift is significant (usually begins by the first half of December as seen in Figure 3), and it introduces a detuning with respect to the timings used at MCDA in the MaxMeanEVI image. This results in a value lower than Amp, which leads to zero for condition B in Figure 2. An additional cause of misidentification (4 over 47 pixels) came from double crop system practices in which maize came after soybean. For maize, the crop cycle is shorter, and there are a variety of choices for short-period cultivars. Therefore, the displacement of the sowing date for soybean is smaller. MCDA performs well with a single crop soybean system; even in the cases when farmers plant two successive crops and thus have to anticipate the soybean sowing time, the model performs fairly well.

As expected, we noticed that the soybean field size caused considerable differences in the classification results. MCDA generated better results where crop fields were larger than the area in which the MCDA was developed, the state of Rio Grande do Sul. Fields with smaller areas were more subject to errors than those with larger areas; this is consistent with a previous analysis in Mato Grosso [17] and also with work from [8, 18].

**Figure 6:**
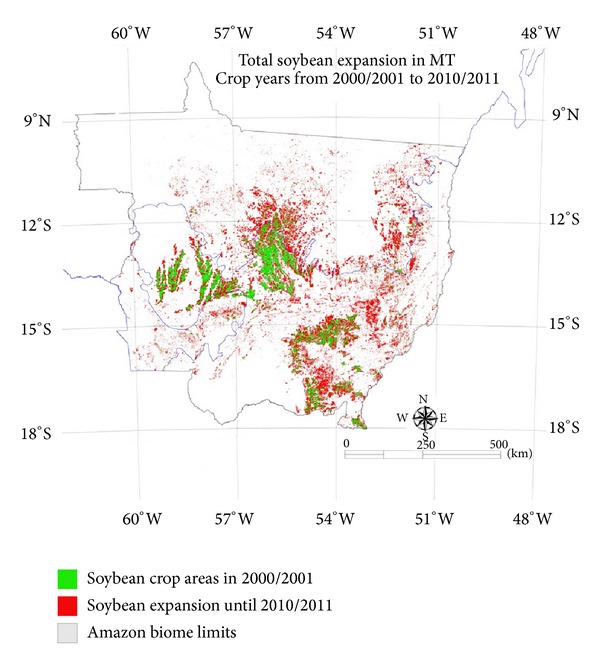
Total soybean crop area expansion in Mato Grosso state after 2000/2001.

An accurate annual soybean crop area map is an efficient tool for surveying the deforestation drivers linked to soybean cultivation in this region.  Figure 6 shows the total area expansion of soybean in red; the overall trend is for expansion toward the north-northeast of Mato Grosso, which was also observed in [17, 19, 29], causing pressure on the Amazon biome.

By comparing MCDA maps from 2001 to 2011, we obtained the total soybean crop area in Mato Grosso.  Figure 6 shows a map of the total soybean crop area with 11,544,000 ha of soybean crops and it demonstrates the expansion of the soybean crop area during the studied period.

## 5. Conclusions 

The MCDA procedure is based on a consistent and objective methodology for estimating soybean crop areas using moderate resolution imaging spectroradiometer (MODIS) images and Enhanced Vegetation Index (EVI) data in the Mato Grosso state. By using predefined parameters, MCDA provides the evolution of annual thematic soybean maps, directly forecasting the soybean cropping areas and area expansion in the state. This is a timesaving procedure and is independent of analyst skills and image interpretation. Our results indicate that, when compared to current official methods for soybean area estimation in Brazil, the MCDA procedure provides a reduction in analysis time, and it is a simple and effective method for providing spatial information.

The total soybean crop expansion area that intrudes into the Amazon biome for the study period (3,463,000 ha) represents more than 55% in Mato Grosso and approximately 14.3% of the total soybean area cultivated in Brazil, based on the crop area for the 2010/2011 crop year.

Implementing operational crop monitoring systems on large areas such as Brazil remains a challenge because it requires accounting for the spatiotemporal variability of environmental conditions and agricultural practices. In this paper, we assessed the efficiency of the MODIS crop detection algorithm (MCDA), which had initially been validated in Southern Brazil, for mapping soybean crop areas in the Amazonian state of Mato Grosso. We validated our approach for two states: Rio Grande do Sul and Mato Grosso (present paper). Additional research would need to be carried out to apply this method to all of Brazil. However, Mato Grosso and Rio Grande do Sul are two important study areas because they present the most extreme cases for cultivating soybean (specifically, different ecoregions, different calendars, and different cropping systems). Therefore, our results suggest that the MCDA is likely to be successful in other regions as well.

## Figures and Tables

**Table 1:** Confusion matrix from the comparison of MCDA and field campaign mapping.

MCDA	Reference (pixels)			
	Soybean	Non-soybean	Total classified	User's accuracy
Soybean	**153**	0	153	**100%**
Non-soybean	47	**200**	250	**76.50%**
Reference total	200	200	**400**	
Producer's accuracy	**80.97%**	**100%**		
**Overall accuracy**	**88.25%**			
**Kappa Index**	**0.765**			
